# Diverging global incidence trends of early-onset cancers: comparisons with incidence trends of later-onset cancers and mortality trends of early-onset cancers

**DOI:** 10.1186/s40779-025-00670-8

**Published:** 2025-11-14

**Authors:** Miyu Terashima, Kota Nakayama, Sora Shirai, Satoko Ugai, Hwa-Young Lee, Haruna Matsui, Hiroki Mizuno, Shiori Tanaka, Minkyo Song, Naoko Sasamoto, Ichiro Kawachi, Edward L. Giovannucci, Tomotaka Ugai

**Affiliations:** 1 Division of Integrative Cancer Research, National Cancer Center Research Institute, Tokyo, 104-0045 Japan; 2 Present Address: Okayama University Medical School, Okayama, 700-8558 Japan; 3 Present Address: Department of Social and Behavioral Sciences, Harvard T.H. Chan School of Public Health, Boston, MA 02215 USA; 4 Present Address: Department of Electrical Engineering and Computer Science, Massachusetts Institute of Technology, Cambridge, MA 02139 USA; 5 Present Address: Department of Epidemiology, Harvard T.H. Chan School of Public Health, Boston, MA 02115 USA; 6 Graduate School of Public Health and Healthcare Management, the Catholic University of Korea, Seoul, 06591 Republic of Korea; 7 Present Address: Catholic Institute for Public Health and Healthcare Management, the Catholic University of Korea, Seoul, 06591 Republic of Korea; 8 Division of Prevention, Institute for Cancer Control, National Cancer Center, Tokyo, 104-0045 Japan; 9 Laboratory of Epidemiology and Population Sciences, National Institute On Aging, National Institutes of Health, Baltimore, MD 21224 USA; 10 Public Health Sciences Division, Fred Hutchinson Cancer Center, Seattle, WA 98109 USA; 11 Department of Nutrition, Harvard T.H. Chan School of Public Health, Boston, MA 02115 USA; 12 Department of Pathology, Brigham and Women’s Hospital, and Harvard Medical School, Boston, MA 02115 USA; 13 Zhu Family Center for Global Cancer Prevention, Harvard T.H. Chan School of Public Health, Boston, MA 02115 USA

**Keywords:** Neoplasms, Epidemiology, Risk factors, Global health, Young adults

## Abstract

**Background:**

The global increase in the incidence of early-onset cancers (defined as cancers diagnosed at 20–49 years old) is a serious public health problem. We investigated 1) whether the incidence trend of early-onset cancers differs from that of later-onset cancers and 2) whether both the incidence and mortality of early-onset cancers have increased concurrently.

**Methods:**

We utilized age-standardized incidence and mortality rates for early-onset and later-onset cancers diagnosed between 2000 and 2017 from the Cancer Incidence in Five Continents and World Health Organization (WHO) mortality databases. The national obesity prevalence among adults aged 20–49 years was obtained from the National Clinical Database. Using joinpoint regression models, we calculated average annual percentage changes (AAPCs) for cancer incidence and mortality by cancer types and countries. We additionally conducted human development index (HDI)-stratified analyses and assessed the correlation between the obesity prevalence in younger populations and early-onset cancer incidence by country. To investigate the more recent trend of early-onset cancer mortality, we extended our mortality analysis after 2017 for cancer types and countries with statistically significant positive AAPCs in both incidence and mortality of early-onset cancers between 2000 and 2017.

**Results:**

Our analysis showed that 10 early-onset cancer types (thyroid cancer, breast cancer, melanoma, uterine cancer, colorectal cancer, kidney cancer, cervical cancer, pancreatic cancer, multiple myeloma, Hodgkin lymphoma) in females and 7 early-onset cancer types (thyroid cancer, kidney cancer, testis cancer, prostate cancer, colorectal cancer, melanoma, leukemia) in males had statistically significant positive AAPCs in at least 10 countries. Among these, the following early-onset cancer types had significantly higher AAPCs than later-onset cancer types in females: colorectal cancer (6 countries; AAPC range: 1.8–3.8%), cervical cancer (6 countries; AAPC range: 1.2–3.3%), pancreatic cancer (5 countries; AAPC range: 2.3–13.0%), and multiple myeloma (5 countries; AAPC range: 3.1–9.8%); in males: prostate cancer (12 countries; AAPC range: 3.9–18.4%), colorectal cancer (8 countries; AAPC range: 1.8–3.2%), and kidney cancer (6 countries; AAPC range: 2.0–6.0%). We observed statistically significant positive AAPCs in both the incidence and mortality of the following early-onset cancer types: uterine cancer (5 countries) and colorectal cancer (3 countries in females and 5 countries in males). The steeper increases in early-onset cancers compared with later-onset cancers were mainly observed in the very high-HDI country group, including early-onset colorectal cancer (AAPC = 2.4%, 95% CI 2.1–2.6 in females; AAPC = 2.0%, 95% CI 1.7–2.4 in males) to later-onset colorectal cancer (AAPC = −0.1%, 95% CI −0.2 to 0 in females; AAPC = −0.2%, 95% CI −0.3 to 0 in males). We observed strong positive correlations between the increasing obesity prevalence and the rising incidence of early-onset obesity-related cancers in several countries, including Australia (7 cancer types), United Kingdom (7 cancer types), Canada (7 cancer types), Republic of Korea (7 cancer types), and USA (6 cancer types) in females and United Kingdom (7 cancer types), Canada (6 cancer types), Australia (5 cancer types), Sweden (5 cancer types), and Republic of Korea (4 cancer types) in males. Although we did not observe an apparent spike after 2017 in many countries, we observed continued increases in the mortality of certain cancer types, such as uterine cancer (Japan, Republic of Korea, United Kingdom, USA, and Ecuador) in females and colorectal cancer (Argentina, Canada, United Kingdom, and USA) in males.

**Conclusions:**

The increase in many early-onset cancer types was significantly higher than that of later-onset cancers, and the incidence and mortality of certain early-onset cancer types (such as colorectal cancer) increased simultaneously. Our study highlights global differences in cancer incidence and mortality trends of early-onset and later-onset cancers.

**Supplementary Information:**

The online version contains supplementary material available at 10.1186/s40779-025-00670-8.

## Background

Early-onset cancers, commonly defined as cancers diagnosed at 20–49 years old, have become an emerging global health concern [1–6]. Over the past few decades, a growing number of countries, especially those in North America, Europe, and Oceania, have reported an increase in early-onset cancers in a variety of organs, including the breast, colorectum, endometrium, esophagus, gallbladder, bile duct, head and neck, kidney, liver, bone marrow (multiple myeloma), pancreas, prostate, stomach, and thyroid [1–6]. In addition to the morbidity and mortality directly caused by these cancers, early-onset cancer patients experience a higher burden of long-term health complications, such as psychological disorders, secondary cancers, cardiovascular disease, and endocrine disorders, compared to later-onset cancer patients [5, 7]. The socioeconomic impact of early-onset cancers also extends beyond direct medical costs, affecting educational attainment, employment prospects, and overall quality of life for affected individuals [5, 8]. Given the clinical and public health burden posed by early-onset cancers, there is an urgent need for a comprehensive understanding of recent global trends.

Emerging evidence suggests that various factors, including lifestyle changes and environmental exposures, may contribute to the rise in early-onset cancers [5, 9, 10]. Although the rising incidence of early-onset cancers is likely partially attributable to the increasing uptake of clinically in-significant cancers by screening and early detection, the increasing incidence of certain early-onset cancer types (especially those with increasing mortality) does not appear to be fully explained by the increased uptake by screening practices and early detection [5, 6]. Conversely, some early-onset cancers, such as thyroid and prostate cancers, have shown rising incidence without a corresponding increase in mortality, suggesting that earlier detection and over-detection of clinically in significant cancers through screening may account for or contribute to the observed trends [5, 6]. Thus, differentiating these patterns is critical to better understand the mechanisms behind the global increase in early-onset cancers and design more effective prevention strategies.

This study aims to provide a comprehensive overview of global cancer trends, utilizing global databases. Specifically, this study aims to investigate: 1) whether the incidence of early-onset cancers has increased concurrently with that of later-onset cancers, and 2) whether the incidence of early-onset cancers has risen in tandem with mortality trends. By exploring these trends, we aim to provide valuable insights into the global rise of early-onset cancers, and spur further research on various early-onset cancer types. This study was motivated by our research hypothesis: “Is the increase in early-onset cancers mainly driven by shifts in risk factor exposure (such as obesity, diets, and novel risk factors) among younger generations?”

## Methods

### Data sources

We obtained age-standardized rates (ASRs) of the incidence of early-onset cancers (diagnosed between ages 20 and 49) and later-onset cancers (diagnosed at age 50 and above) from the Cancer Incidence in Five Continents database, published by the International Agency for Research on Cancer. Detailed descriptions of the original data sources and methodologies used to compile the data can be found on the Cancer Incidence in Five Continents website (https://ci5.iarc.fr). ASRs of the mortality of early-onset cancers were retrieved from the World Health Organization (WHO) mortality database (https://www.who.int/data/data-collection-tools/who-mortality-database). We used these databases because they provide high-quality standardized data on cancer incidence and mortality to estimate cancer burden at national and global levels. To assess the recent trends in the incidence and mortality of early-onset and later-onset cancers, we defined the study period as 2000–2017, based on the availability of the most recent data on cancer incidence in many countries. To investigate the most recent trend of early-onset cancer mortality, we further extended our mortality analysis from 2000 to 2023 (or 2021/2022 depending on data availability).

We retrieved ASRs of early-onset and later-onset cancer incidence in the following 44 countries with available incidence data during the study period: Argentina, Australia, Austria, Bahrain, Belarus, Canada, Chile, China, Colombia, Costa Rica, Croatia, Cyprus, Czech Republic, Denmark, Ecuador, Estonia, Finland, France, Germany, Iceland, India, Ireland, Israel, Italy, Japan, Kuwait, Latvia, Lithuania, Malta, Netherlands, New Zealand, Norway, Philippines, Poland, Republic of Korea, Slovenia, Spain, Sweden, Switzerland, Thailand, Turkey, Uganda, United Kingdom, and USA. Among these countries, data on early-onset cancer mortality were not available in the following 8 countries: Bahrain, Belarus, China, Iceland, India, Thailand, Turkey, and Uganda. Therefore, we included the remaining 36 countries for comparative analyses of incidence and mortality.

We analyzed the following cancer types with available data in most countries: lip, oral cavity and pharynx cancer (ICD-10: C00–14), esophageal cancer (C15), stomach cancer (C16), colorectal cancer (C18–21), liver cancer (C22), gallbladder and extrahepatic bile duct cancer (C23–24), pancreatic cancer (C25), larynx cancer (C32), lung cancer (C33–34), melanoma of skin (C43), breast cancer (C50), cervical cancer (C53), uterine cancer (C54), ovarian cancer (C56), prostate cancer (C61), testis cancer (C62), kidney cancer (C64), bladder cancer (C67), brain and central nervous system cancer (C70–72), thyroid cancer (C73), Hodgkin lymphoma (C81), non-Hodgkin lymphoma (C82–86, C96), multiple myeloma (C88–90), and leukemia (C91–95).

We also retrieved national obesity prevalence data from 2000 to 2017 from the National Clinical Database [11]. The age-standardized obesity prevalence in the younger population (20–49 years old) in each country was estimated referencing the Segi-Doll World standard population (1966) [12].

In addition, we classified the 44 countries into the following 3 groups based on the human development index (HDI), a composite index based on life expectancy, education, and per-capita income indicators for 2017, developed by the UN Development Programme: medium (HDI 0.550–0.699; India, Uganda, Philippines), high (HDI 0.700–0.799; China, Colombia, Costa Rica, Ecuador, Thailand, Turkey), and very high (HDI ≥ 0.800; remaining countries) [13]. None of the included countries was classified into the HDI-low category (HDI < 0.550).

### Statistical analysis

The ASRs of cancer incidence and mortality per 100,000 person-years by cancer types were calculated using direct age standardization referencing the Segi-Doll World standard population (1966) [12]. To evaluate changes in cancer incidence and mortality over time, we calculated average ASRs and average annual percentage changes (AAPCs) with 95% confidence intervals (CIs) during the study period (from 2000 to 2017) for individual cancer types using Joinpoint Regression Program (version 5.0.2) [14]. In line with the recommendations of this program, a maximum of 3 joinpoints (i.e., 4 linear segments) was allowed based on the 18 annual data points (2000–2017) [15]. To determine the optimal number of joinpoints, we used the weighted Bayesian Information Criterion, a data-driven approach that balances model fit and complexity. The Monte Carlo Permutation method was used to assess differences between each segment. Data on missing values in ASRs were excluded from this analysis. Statistical significance corresponded to a 95% CI of AAPC that did not include zero. In other words, if a 95% CI of AAPC included zero, we considered that AAPC as not statistically significant. Because we focused on statistically significant positive AAPCs, we combined non-significant AAPCs and significant negative AAPCs into the same group. Differences in the AAPCs between early-onset and later-onset cancer incidence were assessed by comparing 95% CIs, where non-overlapping 95% CIs were considered statistically significantly different.

To account for sociodemographic, lifestyle, and environmental factors, we additionally conducted HDI-stratified analyses and assessed the correlation between the obesity prevalence in younger populations and early-onset cancer incidence by country. We calculated Spearman’s rank correlation coefficient (*ρ*) as a measure of correlation. All *P*-values were two-sided and a *P* < 0.05 was considered statistically significant; however, these analyses are secondary and exploratory analyses, and the results should be interpreted cautiously.

## Results

### Comparison of early-onset and later-onset cancer incidence

**Fig. 1:**
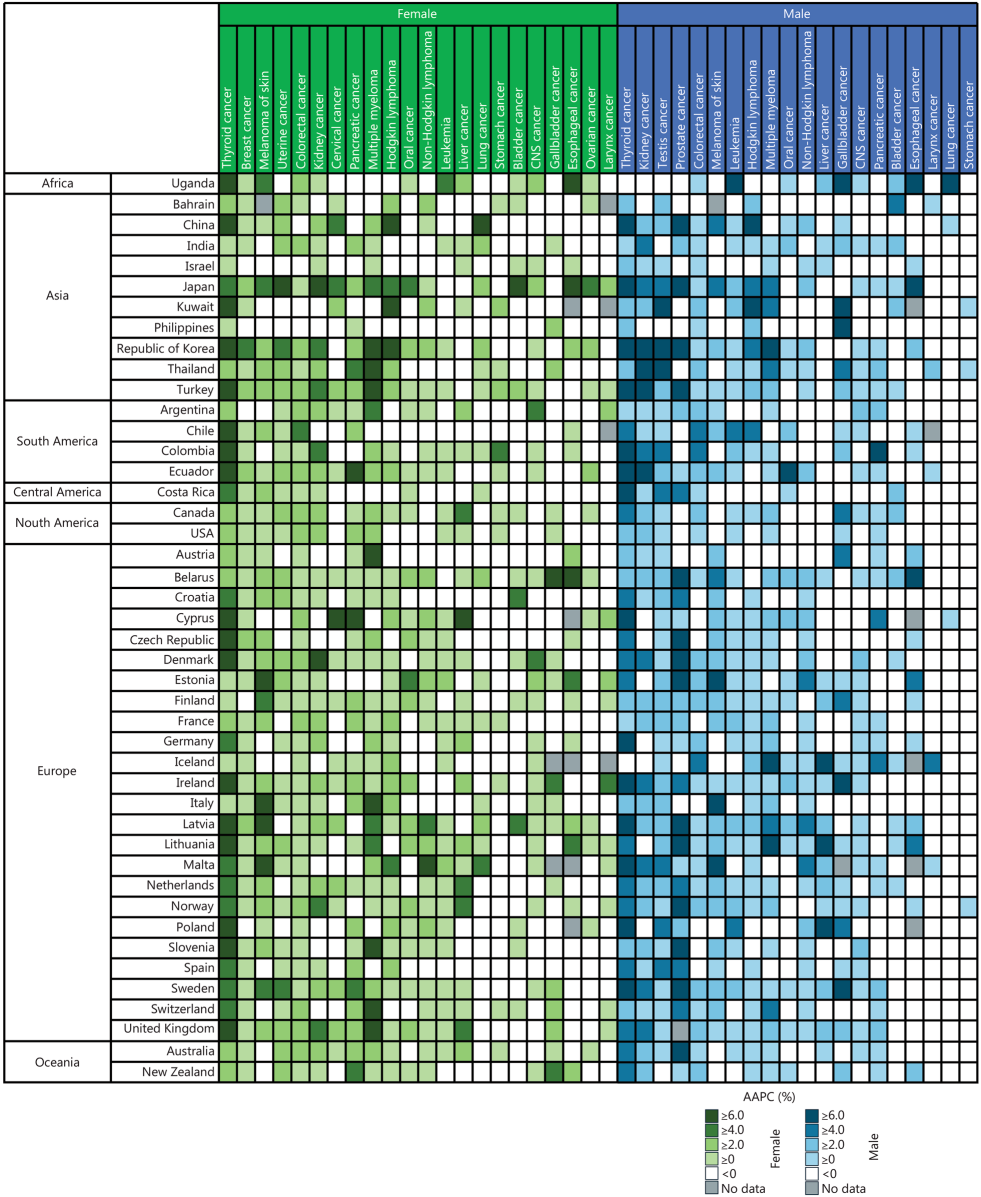
Magnitude of increases in early-onset cancer incidence. Gallbladder cancer includes gallbladder and extrahepatic bile duct cancers. CNS cancer includes brain and central nervous system (CNS) cancer. Oral cancer includes lip, oral cavity, and pharynx cancers. AAPC average annual percentage change

**Fig. 2:**
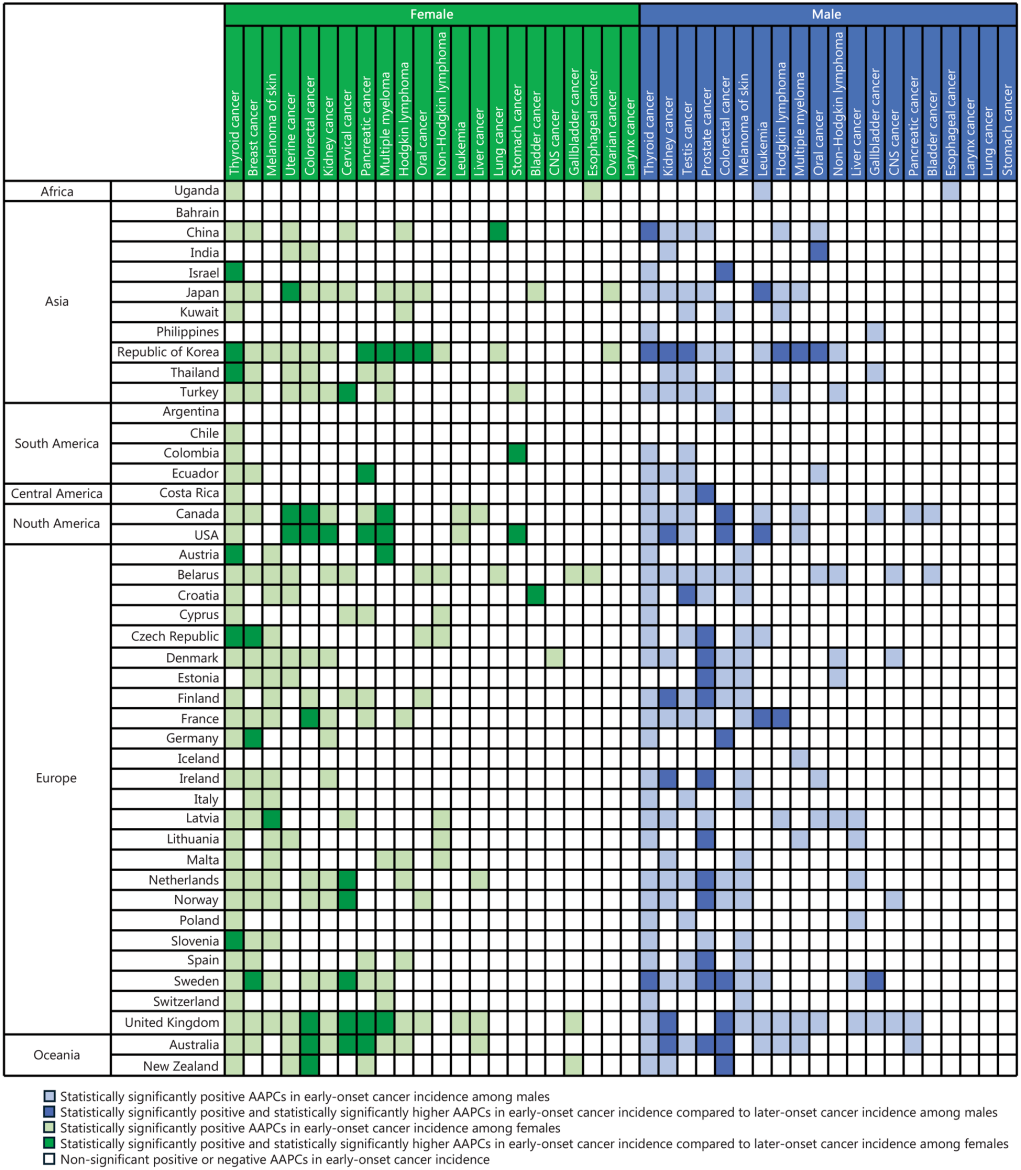
Comparisons between the early-onset and later-onset cancer incidence trends. Gallbladder cancer includes gallbladder and extrahepatic bile duct cancers. CNS cancer includes brain and central nervous system (CNS) cancer. Oral cancer includes lip, oral cavity, and pharynx cancers. AAPC average annual percentage change

For early-onset cancers in females, thyroid cancer exhibited the highest number of countries (37 out of 44 countries) with statistically significant positive AAPCs, followed by breast cancer (23 countries), melanoma (19 countries), uterine cancer (17 countries), and colorectal cancer (16 countries) (Figs. 1 and 2**; **Additional file 1: Table S1). Among the countries with significant positive AAPCs, the range varied from 1.1% (95% CI 0.2–2.1) in Finland to 17.7% (95% CI 15.8–20.0) in China for thyroid cancer, from 0.5% (95% CI 0.2–0.7) in Australia to 5.4% (95% CI 5.1–5.8) in Republic of Korea for breast cancer, 1.3% (95% CI 0.5–2.3) in Austria to 9.1% (95% CI 1.3–17.7) in Malta for melanoma, and 1.4% (95% CI 0.4–2.3) in Finland to 3.8% (95% CI 3.2–4.4) in United Kingdom for colorectal cancer (Additional file 1: Table S1). Among these early-onset cancer types in females with statistically significant positive AAPCs, we observed statistically significantly higher AAPCs in early-onset cancers than in later-onset cancers in the following cancer types: colorectal cancer (6 countries; Canada, USA, France, United Kingdom, Australia, New Zealand), cervical cancer (6 countries; Turkey, the Netherlands, Norway, Sweden, United Kingdom, Australia), pancreatic cancer (5 countries; Republic of Korea, Ecuador, USA, United Kingdom, Australia), and multiple myeloma (5 countries; Republic of Korea, Canada, USA, Austria, United Kingdom) (Fig. 2). These faster increases in early-onset than in later-onset cancers across multiple cancer types, were mainly observed globally, especially in North America, Europe, and Oceania (Additional file 1: Table S2). A sharper increase in early-onset colorectal cancer (AAPC = 2.4%, 95% CI 2.1–2.6) compared to later-onset colorectal cancer (AAPC = −0.1%, 95% CI −0.2 to 0) was observed in the very high-HDI country group (Additional file 1: Table S3).

For early-onset cancers in males, thyroid cancer showed the highest number of countries (34 out of 44 countries) with statistically significant positive AAPCs, followed by kidney cancer (22 countries), testis cancer (22 countries), prostate cancer (21 countries), and colorectal cancer (18 countries) (Figs. 1 and 2**; **Additional file 1: Table S4). Among the countries with significant positive AAPCs, the range for thyroid cancer varied from 1.9% (95% CI 0.7–3.1) in France to 20.7% (95% CI 18.4–22.5) in China. For kidney cancer, the range was from 2.0% (95% CI 1.0–3.0) in Finland to 9.2% (95% CI 3.1–15.3) in Ecuador. Similarly, for testis cancer, the range was from 0.4% (95% CI 0.1–0.8) in USA to 7.5% (95% CI 1.6–13.9) in Kuwait (Additional file 1: Table S4). Among these early-onset cancer types in males with statistically significant positive AAPCs, we observed statistically significantly higher AAPCs in early-onset cancers than in later-onset cancers in the following cancer types: prostate cancer (12 countries; Costa Rica, Czech Republic, Denmark, Estonia, Finland, Ireland, Lithuania, the Netherlands, Norway, Spain, Sweden, Australia), colorectal cancer (8 countries; Israel, Canada, USA, Germany, Sweden, United Kingdom, Australia, New Zealand), and kidney cancer (6 countries; Republic of Korea, USA, Finland, Ireland, United Kingdom, Australia) (Fig. 2). A sharper increase in early-onset colorectal cancer than later-onset colorectal cancer was observed globally, especially in North America, Europe, Oceania (Additional file 1: Table S2). When stratified by the HDI, these steeper increases in early-onset cancers compared with later-onset cancers were mainly observed in the very high-HDI country group (Additional file 1: Table S3), including early-onset colorectal cancer (AAPC = 2.0%, 95% CI 1.7–2.4) compared to later-onset colorectal cancer (AAPC = −0.2%, 95% CI −0.3 to 0), early-onset prostate cancer (AAPC = 2.2%, 95% CI 1.9–2.6) compared to later-onset prostate cancer (AAPC = −0.4%, 95% CI −1.0 to 0.2), and early-onset kidney cancer (AAPC = 3.5%, 95% CI 3.0–3.9) compared to later-onset kidney cancer (AAPC = 2.1%, 95% CI 1.8–2.4).

Detailed data on ASRs and AAPCs in early-onset and later-onset cancer incidence are described in Additional file 1: Tables S1 and S4.

### Correlation between obesity prevalence and early-onset cancer incidence

We assessed the correlations between obesity prevalence in younger populations and incidence of early-onset obesity-related cancers in countries with statistically significant positive AAPCs (Additional file 2: Figs. S1–S11). Overall, our results showed strong positive correlations between the increasing obesity prevalence and the rising incidence of early-onset obesity-related cancers in many countries. In females, we observed statistically significant positive correlations for thyroid cancer in 32 countries (China, Israel, Japan, Republic of Korea, Thailand, Turkey, Chile, Colombia, Ecuador, Costa Rica, Canada, USA, Austria, Croatia, Cyprus, Czech Republic, Denmark, Finland, Germany, Ireland, Latvia, Lithuania, Malta, Netherlands, Norway, Poland, Slovenia, Sweden, Switzerland, United Kingdom, Australia, New Zealand), followed by uterine cancer (16 countries; China, India, Japan, Republic of Korea, Thailand, Turkey, Ecuador, Canada, USA, Croatia, Denmark, Estonia, Lithuania, United Kingdom, Australia, New Zealand), kidney cancer (13 countries; Japan, Republic of Korea, Turkey, Canada, USA, Denmark, Germany, Ireland, Netherlands, Norway, Sweden, United Kingdom, Australia), colorectal cancer (11 countries; Japan, Republic of Korea, Thailand, Turkey, Canada, USA, Denmark, Finland, Sweden, United Kingdom, Australia), and multiple myeloma (10 countries; Japan, Republic of Korea, Thailand, Turkey, Canada, USA, Malta, Sweden, United Kingdom, Australia). At a country level, significant positive correlations for many cancer types were observed in several countries, especially in Australia (7 cancer types; thyroid cancer, colorectal cancer, uterine cancer, kidney cancer, pancreatic cancer, multiple myeloma, liver cancer), United Kingdom (7 cancer types; thyroid cancer, colorectal cancer, uterine cancer, kidney cancer, pancreatic cancer, multiple myeloma, liver cancer), Canada (7 cancer types; thyroid cancer, colorectal cancer, uterine cancer, kidney cancer, pancreatic cancer, multiple myeloma, liver cancer), Republic of Korea (7 cancer types; thyroid cancer, colorectal cancer, uterine cancer, kidney cancer, pancreatic cancer, multiple myeloma, ovarian cancer), and USA (6 cancer types; thyroid cancer, colorectal cancer, uterine cancer, kidney cancer, pancreatic cancer, multiple myeloma, stomach cancer). Significant negative correlations were observed in the following cancer types and countries: thyroid cancer (Kuwait, Belarus, and Spain), and pancreatic cancer (Spain), which were reflected in recent decreasing trends in obesity. In males, we observed statistically significant positive correlations for thyroid cancer in 27 countries (China, Israel, Japan, Republic of Korea, Colombia, Ecuador, Costa Rica, Canada, Austria, Belarus, Croatia, Cyprus, Denmark, Finland, Ireland, Italy, Latvia, Lithuania, Netherlands, Norway, Poland, Slovenia, Sweden, Switzerland, United Kingdom, Australia, New Zealand), followed by kidney cancer (20 countries; China, India, Japan, Republic of Korea, Turkey, Ecuador, Canada, USA, Belarus, Denmark, Finland, Ireland, Latvia, Malta, Netherlands, Norway, Sweden, United Kingdom, Australia, New Zealand), colorectal cancer (16 countries; Israel, Republic of Korea, Thailand, Argentina, Canada, USA, Belarus, Denmark, Estonia, Finland, Netherlands, Norway, Sweden, United Kingdom, Australia, New Zealand), multiple myeloma (7 countries; Japan, Republic of Korea, Canada, USA, Lithuania, United Kingdom, Australia), and liver cancer (5 countries; Latvia, Lithuania, Poland, Sweden, United Kingdom). At a country level, significant positive correlations for many cancer types were observed in several countries, including United Kingdom (7 cancer types; thyroid cancer, kidney cancer, colorectal cancer, multiple myeloma, gallbladder cancer, pancreatic cancer, liver cancer), Canada (6 cancer types; thyroid cancer, kidney cancer, colorectal cancer, multiple myeloma, gallbladder cancer, pancreatic cancer), Australia (5 cancer types; thyroid cancer, kidney cancer, colorectal cancer, multiple myeloma, pancreatic cancer), Sweden (5 cancer types; thyroid cancer, kidney cancer, colorectal cancer, gallbladder cancer, liver cancer), Republic of Korea (4 cancer types; thyroid cancer, kidney cancer, colorectal cancer, multiple myeloma).

### Comparison of early-onset cancer incidence and early-onset cancer mortality

**Fig. 3:**
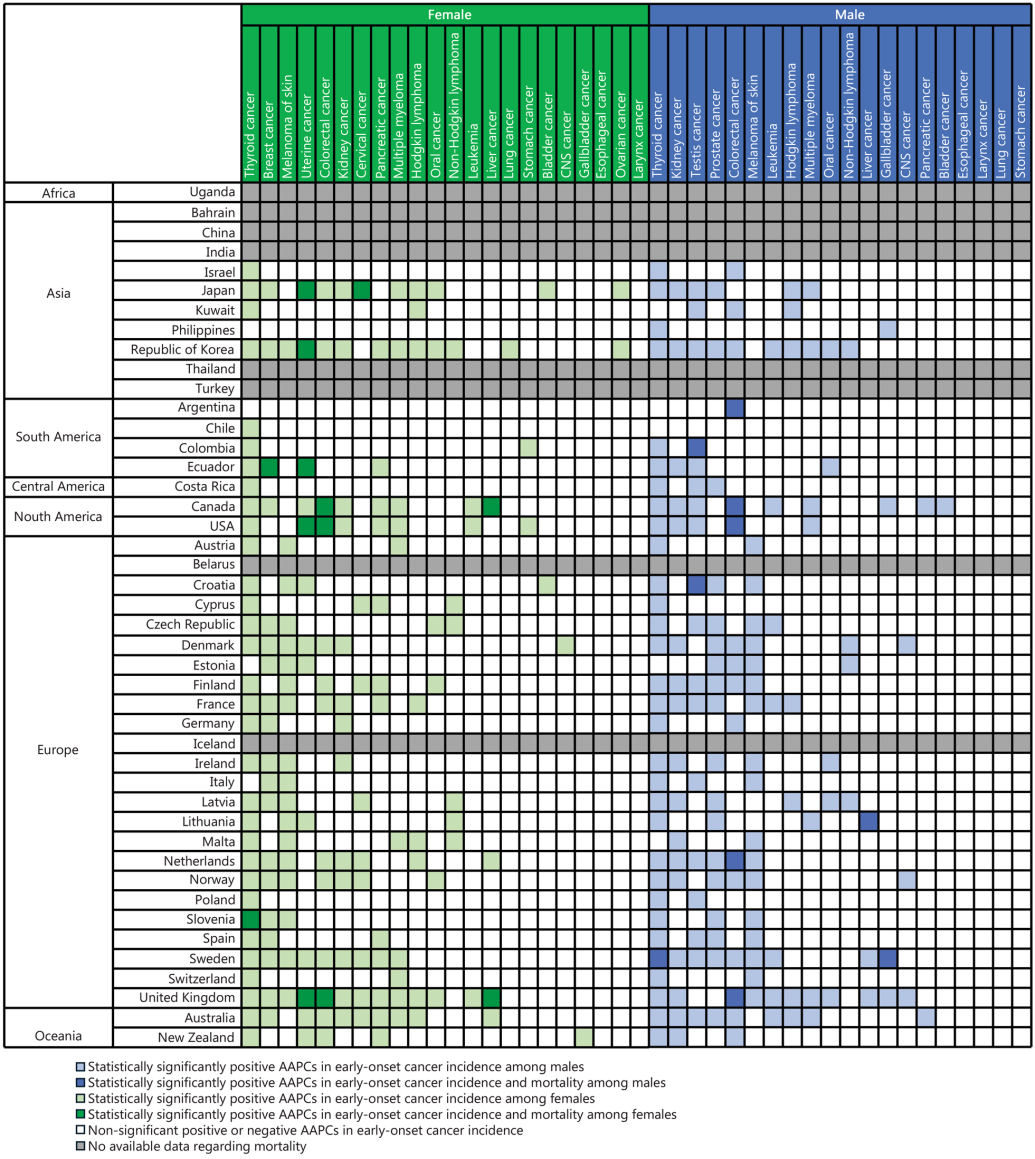
Comparisons between the early-onset cancer incidence trend and the early-onset cancer mortality trend. Gallbladder cancer includes gallbladder and extrahepatic bile duct cancers. CNS cancer includes brain and central nervous system (CNS) cancer. Oral cancer includes lip, oral cavity, and pharynx cancers. AAPC average annual percentage change

In females, although many early-onset cancer types exhibited statistically significant positive AAPCs in incidence in various parts of the world, many of them did not show statistically significant positive AAPCs in mortality. Both the incidence and mortality of early-onset cancer showed statistically significant positive AAPCs in the following cancer types and countries: uterine cancer (Japan, Republic of Korea, Ecuador, USA, and United Kingdom), colorectal cancer (Canada, USA, and United Kingdom), liver cancer (Canada, United Kingdom), breast cancer (Ecuador), cervical cancer (Japan), and thyroid cancer (Slovenia) (Fig. 3; Additional file 2: Fig. S12). For uterine cancer, the range of statistically significant positive AAPCs in early-onset cancer mortality varied from 3.1% (95% CI 1.5–4.8) in the Philippines to 19.7% (95% CI 10.6–29.3) in Ecuador. For early-onset colorectal cancer mortality, the range was 0.5% (95% CI 0.1–1.0) in the USA to 3.3% (95% CI 2.7–4.0) in the Philippines (Additional file 1: Table S5).

Similarly, in males, we did not observe statistically significant positive AAPCs in the mortality of many early-onset cancer types. Both the incidence and mortality of early-onset cancer showed statistically significant positive AAPCs in the following cancer types and countries: colorectal cancer (Argentina, Canada, USA, Netherlands, and United Kingdom), testis cancer (Colombia and Croatia), liver cancer (Lithuania), gallbladder cancer (Sweden), and thyroid cancer (Sweden) (Fig. 3; Additional file 2: Fig. S13). For early-onset colorectal cancer mortality, statistically significant positive AAPC ranged from 0.6% (95% CI 0.2–0.9) in the USA to 3.6% (95% CI 1.5–5.8) in Chile (Additional file 1: Table S6).

To investigate the more recent trend of early-onset cancer mortality, we extended our mortality analysis after 2017 for cancer types and countries with statistically significant positive AAPCs in both incidence and mortality of early-onset cancers from 2000 to 2023 (or 2021/2022 depending on data availability). Although we did not observe an apparent spike after 2017 in many countries, we observed continued increases in the mortality of certain cancer types, such as uterine cancer [Japan (AAPC = 2.8%, 95% CI 1.7–4.2), Republic of Korea (AAPC = 6.3%, 95% CI 4.4–8.6), United Kingdom (AAPC = 4.0%, 95% CI 2.5–5.7), USA (AAPC = 3.8%, 95% CI 2.6–4.9), and Ecuador (AAPC = 12.8%, 95% CI 4.6–21.8)] in females and colorectal cancer [Argentina (AAPC = 1.0%, 95% CI 0.6–1.4), Canada (AAPC = 0.9%, 95% CI 0.3–1.6), United Kingdom (AAPC = 1.2%, 95% CI 0.6–1.7), and USA (AAPC = 0.5%, 95% CI 0.3–0.9)] in males (Additional file 1: Table S7; Additional file 2: Figs. S14 and S15).

## Discussion

Our study highlights global differences in cancer incidence trends between early-onset and later-onset cancers. Notably, in North America, Oceania, and parts of Europe, the incidence of certain early-onset types, such as colorectal cancer, kidney cancer, uterine cancer, and multiple myeloma, showed more dramatic increases compared to later-onset cancer types. We observed concurrent rises in both incidence and mortality for several early-onset cancer types, including colorectal and uterine cancers, in several countries. The steeper increases in early-onset cancers compared with later-onset cancers were mainly observed in the very high-HDI country group. We also observed strong positive correlations between the increasing obesity prevalence and the rising incidence of early-onset obesity-related cancers in many countries, suggesting the important role of the global obesity epidemic in the early-onset cancer epidemic.

The incidence of early-onset cancers increased in many countries. There are several possible hypotheses for this phenomenon [5, 16–18]. The first hypothesis is that, starting in the mid-twentieth century, risk factor exposure has steadily increased across generations. In this case, we assume that both early-onset and later-onset cancers have increased. The second hypothesis is that exposure to risk factors (including new risk factors) has shifted towards younger generations. In this case, the incidence of early-onset cancer may show more dramatic increases than that of later-onset cancer. This study showed that several early-onset cancer types, such as pancreatic, kidney, and colorectal cancers, had significantly higher AAPCs than later-onset cancer types. Because these cancer types are obesity-related cancers, this finding may reflect a shift in energy balance exposures (e.g., obesity, physical inactivity, and sedentary lifestyles) towards younger generations.

The exact explanation for increasing early-onset cancer trends across various countries remains unknown. However, the temporal changes in many exposures over the past century seem to have contributed to the recent increasing trend of multiple early-onset cancer types. Since the mid-twentieth century, the global trend of exposures, including diet, lifestyle, environment, and microbiota, has changed dramatically, followed by the increasing incidence of early-onset cancers [5, 19–21]. Westernization of diets has paralleled a notable surge in obesity and type 2 diabetes among young populations [22–24]. These patterns suggest that the rise in early-onset cancers may reflect a broader shift in established and/or new cancer risk factors to younger populations. Socioeconomic status also likely plays an important role in various factors, such as diets, environments, lifestyles, and access to health care. Further investigations are warranted to clarify the potential mechanism behind the global rise in early-onset cancers.

We also observed concurrent rises in both incidence and mortality for several early-onset cancer types. Although mortality is a complex outcome, comparisons between incidence and mortality in early-onset cancer can provide valuable insight. Notably, the incidence of several early-onset cancer types has increased concurrently with their corresponding mortality, which does not seem to be explained solely by increased screening practices. By contrast, consistent with another study [6], increasing incidence was observed without a corresponding increase in mortality for certain early-onset cancer types, such as thyroid, prostate, and non-melanoma skin cancers, suggesting that over-detection of clinically insignificant cancers through screening might contribute to the observed trends in these cancer types [25, 26].

The increasing incidence of early-onset colorectal cancer, especially in North America, Europe, and Oceania, was consistently observed in other studies [4, 19, 27–31]. We observed sharper increases in early-onset colorectal cancer than in later-onset colorectal cancer. We also observed concurrent increases in both incidence and mortality for early-onset colorectal cancer in several countries. Our and other findings indicate that the global rise of early-onset colorectal cancer is a genuine upward trend. Globally, nearly 10% of colorectal cancer cases are estimated to occur in individuals under the age of 50 [32]. If the current trends continue, the incidence rates of colorectal cancer are projected to increase by 90% in those aged 20–34 years and by 46% in those aged 35–49 years by 2030 [33]. Tailored prevention and early detection strategies are warranted to plateau and reverse the global increasing trend of early-onset colorectal cancer.

Successful global cancer prevention and control initiatives incorporate both primary prevention and secondary prevention strategies. For instance, global initiatives on comprehensive tobacco control, including taxation, warning labels, advertising bans, and smoke-free environments, have effectively reduced global smoking prevalence [34]. Similarly, national anti-human papillomavirus and anti-hepatitis B virus vaccination programs have led to a significant decrease in cervical cancer and liver cancer in many countries [35–38]. At the same time, the implementation of screening programs that can detect early-onset cancers at a curable stage, especially for high-risk populations, is considerably important. Therefore, in addition to efforts for primary prevention of early-onset cancers, the development of risk- and benefit-based screening programs incorporating genetic, dietary, lifestyle, and environmental risk factors is warranted.

This study has several strengths. This study offers comprehensive international comparisons of early- and later-onset cancer incidence rates as well as early-onset cancer incidence and mortality rates across various cancer types and countries. Our findings provide essential insights into global trends and differences in cancer risk by age of onset. Examining the global trends provides valuable descriptive data that will stimulate further research on early-onset cancers. Additionally, the use of the Cancer Incidence in Five Continents database, which aggregates cancer incidence data from diverse sources worldwide, enables robust, cross-national analyses across a wide range of cancer types and regions, enhancing the study’s relevance and applicability to diverse global populations. This global analysis reveals age-specific patterns that are critical for our understanding of cancer trends on a global scale and provides a foundation for more detailed, mechanistic research in the future.

This study should be interpreted with consideration of the following limitations. First, cancer incidence and mortality data were not available for several regions, especially parts of Asia, Africa, South America, and Central America, which limits the global representativeness. For example, we observed an increasing trend of certain early-onset cancers in China; however, we were not able to conduct a comparative analysis of the incidence and mortality in China. Additionally, although the IARC/WHO ensures the quality of cancer incidence and mortality data, the data quality across cancer registries might vary between countries, which potentially affects the accuracy and comparability of reported rates. Further research using more granular data in more countries and regions is warranted. Second, our study was not able to account for histological or molecular subtypes within cancer types due to data constraints. For instance, adenocarcinoma and squamous cell carcinoma in esophageal cancer are different. Microsatellite instability (MSI)-high colorectal carcinoma also differs from non-MSI-high colorectal carcinoma in terms of etiology and risk factors. Future studies that incorporate data on histopathological and anatomical subtypes could provide more nuanced insights. Third, although we considered the effect of screening and over-detection by comparing early-onset cancer incidence and mortality, we were not able to stratify by stage and directly consider screening rates at the country level. Future stage-stratified analyses or simulation models for screening-adjusted trends can be useful to distinguish true biological increases from detection artifacts. Lastly, to develop personalized preventive and treatment strategies, we need to better understand the etiological mechanisms of early-onset cancers. Multi-omics and immunological analyses of exposures and biospecimens will further improve our understanding. Findings on life-course risk factors will contribute to promoting risk factor avoidance among young people and parents. Importantly, to tackle the global rise in early-onset cancers, international transdisciplinary collaborations are warranted.

## Conclusions

In conclusion, we found that the increase in many early-onset cancer types was significantly higher than that of later-onset cancers. Additionally, we observed that the incidence and mortality of certain early-onset cancer types (such as colorectal cancer) increased simultaneously. Our study also highlights the differences in incidence and mortality patterns of early- and late-onset cancers by regions and socioeconomic status indicating a need for further studies to investigate potential biological, environmental, and lifestyle factors contributing to the rising burden of specific early-onset cancer types.

## Supplementary Information

**Additional file 1. Table S1** Incidence trends of early-onset and later-onset digestive, urinary, reproductive, respiratory, endocrine, nervous, hematopoietic, integumentary, and oral and upper aerodigestive tract system cancers among females by country in 2000 – 2017.** Table S2** Incidence trends of early-onset and later-onset cancers by geographical region in 2000 – 2017 for the top 5 common early-onset cancer types with increasing incidence.** Table S3** Incidence trends of early-onset and later-onset cancers by human development index (HDI) levels for the top 5 common early-onset cancer types with increasing incidence.** Table S4** Incidence trends of early-onset and later-onset digestive, urinary, reproductive, respiratory, endocrine, nervous, hematopoietic, integumentary, and oral and upper aerodigestive tract system cancers among males by country in 2000 – 2017.** Table S5** Incidence and mortality trends of early-onset digestive, urinary, reproductive, respiratory, endocrine, nervous, hematopoietic, integumentary system, and oral and upper aerodigestive tract system cancers among females by country in 2000 – 2017.** Table S6** Incidence and mortality trends of early-onset digestive, urinary, reproductive, respiratory, endocrine, nervous, lymphatic and hematopoietic, integumentary, and oral and upper aerodigestive tract system cancers among males by country in 2000 – 2017.** Table S7** Mortality trends of early-onset cancers from 2000 to 2023 (or 2021/2022 depending on data availability).

**Additional file 2.** **Fig. S1** Correlation between obesity prevalence and the incidence of early-onset thyroid cancer among females.** Fig. S2** Correlation between obesity prevalence and the incidence of early-onset multiple myeloma among females.** Fig. S3** Correlation between obesity prevalence and the incidence of early-onset uterine cancer among females.** Fig. S4** Correlation between obesity prevalence and the incidence of early-onset colorectal cancer among females.** Fig. S5** Correlation between obesity prevalence and the incidence of early-onset kidney cancer among females.** Fig. S6** Correlation between obesity prevalence and the incidence of early-onset pancreatic cancer among females.** Fig. S7** Correlation between obesity prevalence and the incidence of early-onset liver, gallbladder, stomach, esophagus, and ovarian cancer among females.** Fig. S8** Correlation between obesity prevalence and the incidence of early-onset thyroid cancer among males.** Fig. S9** Correlation between obesity prevalence and the incidence of early-onset kidney cancer among males.** Fig. S10** Correlation between obesity prevalence and the incidence of early-onset colorectal cancer among males.** Fig. S11** Correlation between obesity prevalence and the incidence of early-onset multiple myeloma, liver cancer, pancreatic cancer, esophageal cancer, and gall bladder cancer among males.** Fig. S12** Countries with increases in both incidence and mortality of early-onset cancers in females.** Fig. S13** Countries with increases in both incidence and mortality of early-onset cancers in males.** Fig. S14** Mortality trends of early-onset cancers from 2000 to 2023 (or 2021/2022 depending on data availability) in females.** Fig. S15** Mortality trends of early-onset cancers from 2000 to 2023 (or 2021/2022 depending on data availability) in males.

## Data Availability

The datasets supporting the conclusions of this article are available in the Cancer Incidence in Five Continents database (https://ci5.iarc.fr/) and the WHO mortality database (https://www.who.int/data/data-collection-tools/who-mortality-database).

## Abbreviations

AAPC: Average annual percentage change
ASR: Age-standardized rate
CI: Confidence interval
HDI: Human development index
MSI: Microsatellite instability
