# Supplementary material for: Diverging global incidence trends of early-onset cancers: comparisons with incidence trends of later-onset cancers and mortality trends of early-onset cancers
Source: Mil Med Res. 2025 Nov 14;12:79. doi: 10.1186/s40779-025-00670-8 (PMC12616995; doi:10.1186/s40779-025-00670-8)
Supplement: Supplementary file 2 — Additional file 2. Fig. S1 Correlation between obesity prevalence and the incidence of early-onset thyroid cancer among females. Fig. S2 Correlation between obesity prevalence and the incidence of early-onset multiple myeloma among females. Fig. S3 Correlation between obesity prevalence and the incidence of early-onset uterine cancer among females. Fig. S4 Correlation between obesity prevalence and the incidence of early-onset colorectal cancer among females. Fig. S5 Correlation between obesity prevalence and the incidence of early-onset kidney cancer among females. Fig. S6 Correlation between obesity prevalence and the incidence of early-onset pancreatic cancer among females. Fig. S7 Correlation between obesity prevalence and the incidence of early-onset liver, gallbladder, stomach, esophagus, and ovarian cancer among females. Fig. S8 Correlation between obesity prevalence and the incidence of early-onset thyroid cancer among males. Fig. S9 Correlation between obesity prevalence and the incidence of early-onset kidney cancer among males. Fig. S10 Correlation between obesity prevalence and the incidence of early-onset colorectal cancer among males. Fig. S11 Correlation between obesity prevalence and the incidence of early-onset multiple myeloma, liver cancer, pancreatic cancer, esophageal cancer, and gall bladder cancer among males. Fig. S12 Countries with increases in both incidence and mortality of early-onset cancers in females. Fig. S13 Countries with increases in both incidence and mortality of early-onset cancers in males. Fig. S14 Mortality trends of early-onset cancers from 2000 to 2023 (or 2021/2022 depending on data availability) in females. Fig. S15 Mortality trends of early-onset cancers from 2000 to 2023 (or 2021/2022 depending on data availability) in males. [file 40779_2025_670_MOESM2_ESM.pdf]

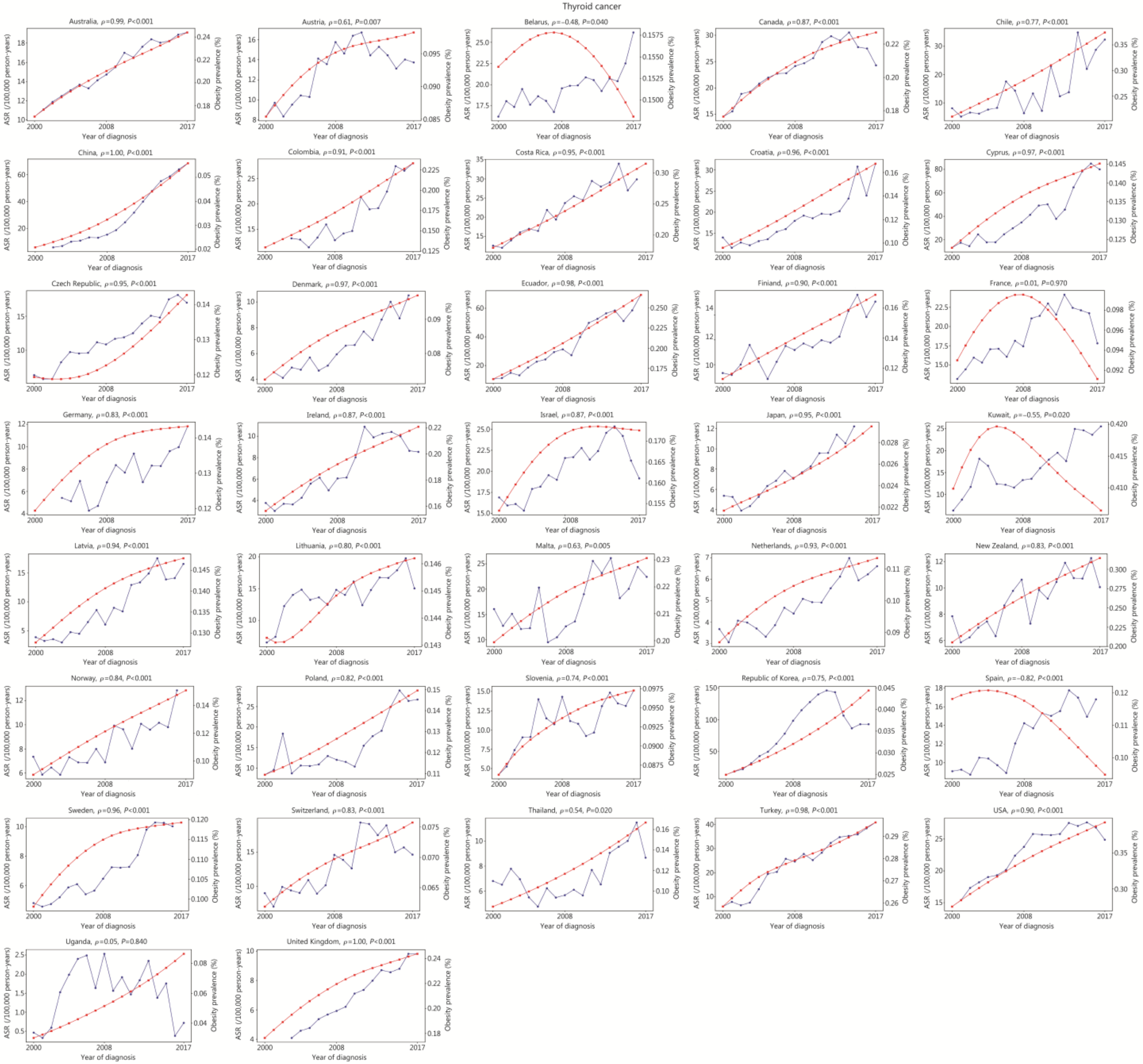

Fig. S1 Correlation between obesity prevalence and the incidence of early-onset thyroid cancer among females. Blue lines show early-onset cancer incidence, and red lines show obesity prevalence among younger populations aged 20 – 49 years from 2000 to 2017. ASR age-standardized rate

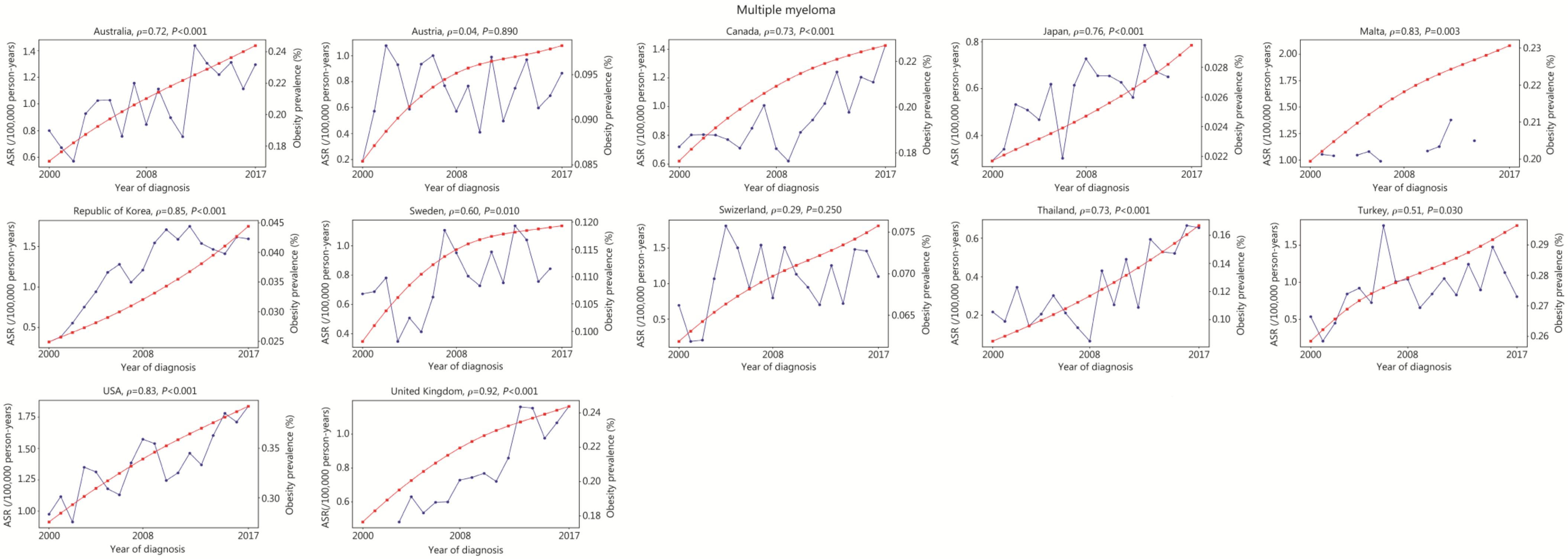

Fig. S2 Correlation between obesity prevalence and the incidence of early-onset multiple myeloma among females. Blue lines show early-onset cancer incidence, and red lines show obesity prevalence among younger populations aged 20 – 49 years from 2000 to 2017. ASR age-standardized rate

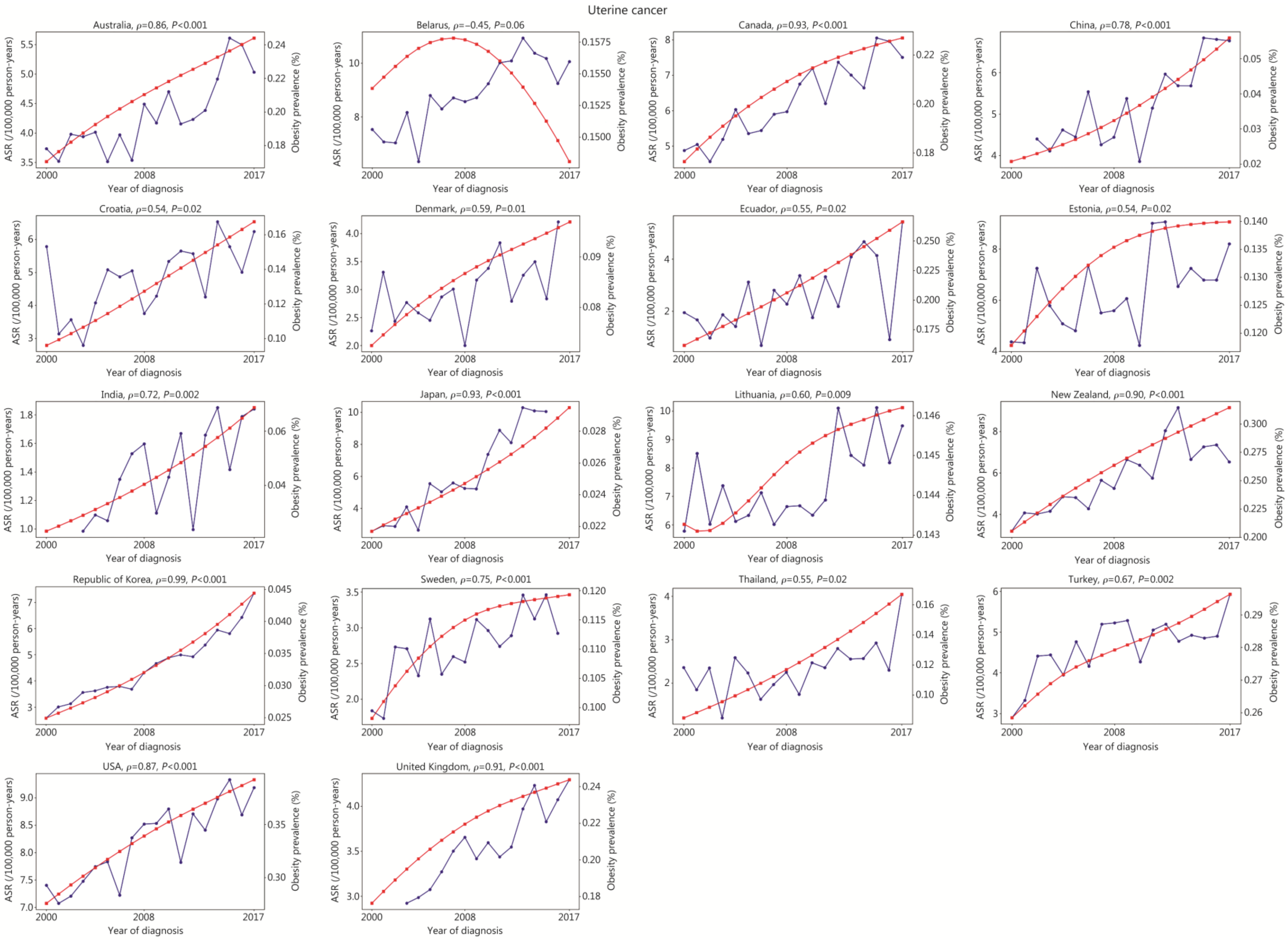

Fig. S3 Correlation between obesity prevalence and the incidence of early-onset uterine cancer among females. Blue lines show early-onset cancer incidence, and red lines show obesity prevalence among younger populations aged 20 – 49 years from 2000 to 2017. ASR age-standardized rate

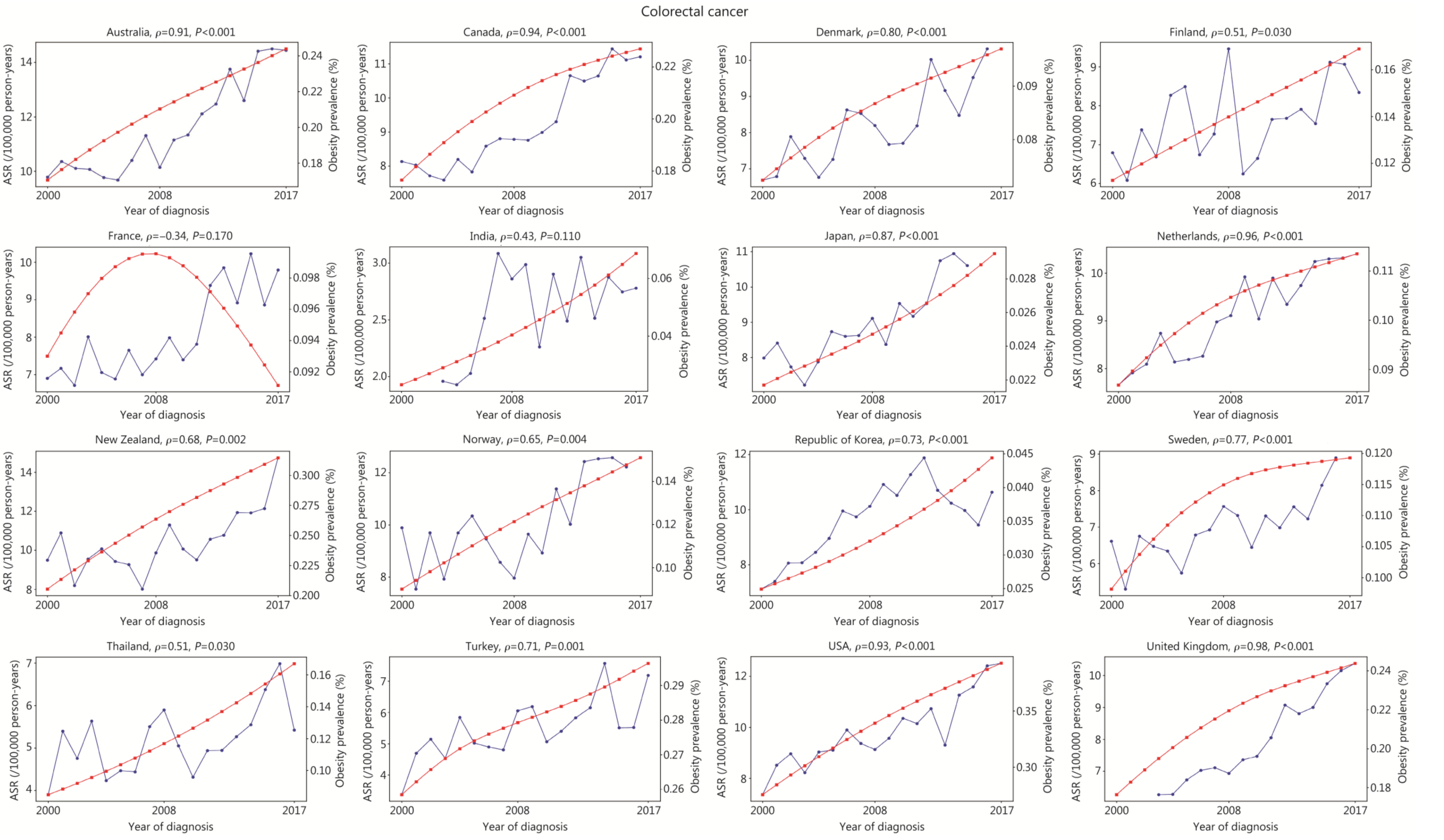

Fig. S4 Correlation between obesity prevalence and the incidence of early-onset colorectal cancer among females. Blue lines show early-onset cancer incidence, and red lines show obesity prevalence among younger populations aged 20 – 49 years from 2000 to 2017. ASR age-standardized rate

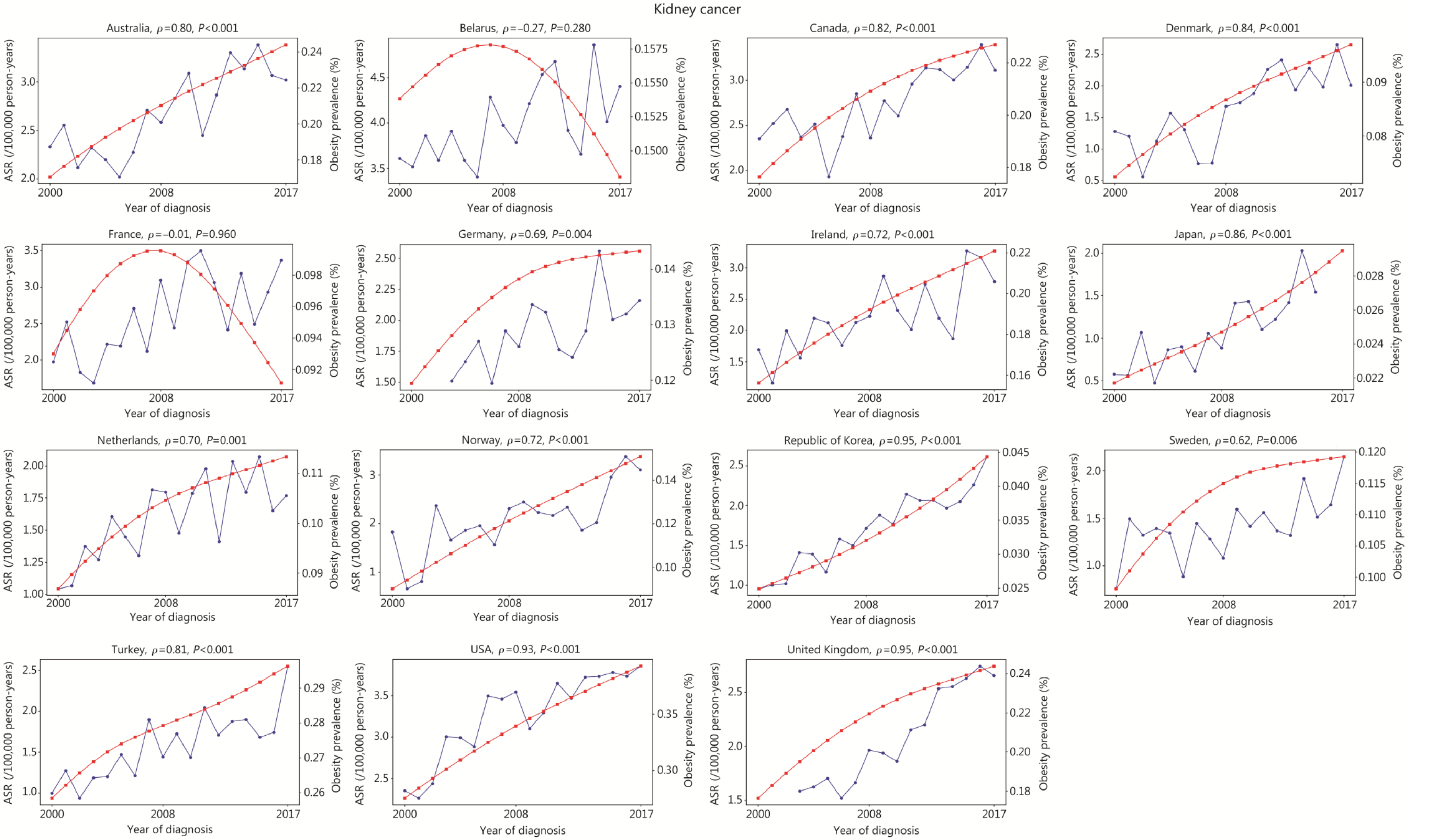

Fig. S5 Correlation between obesity prevalence and the incidence of early-onset kidney cancer among females. Blue lines show early-onset cancer incidence, and red lines show obesity prevalence among younger populations aged 20 – 49 years from 2000 to 2017. ASR age-standardized rate

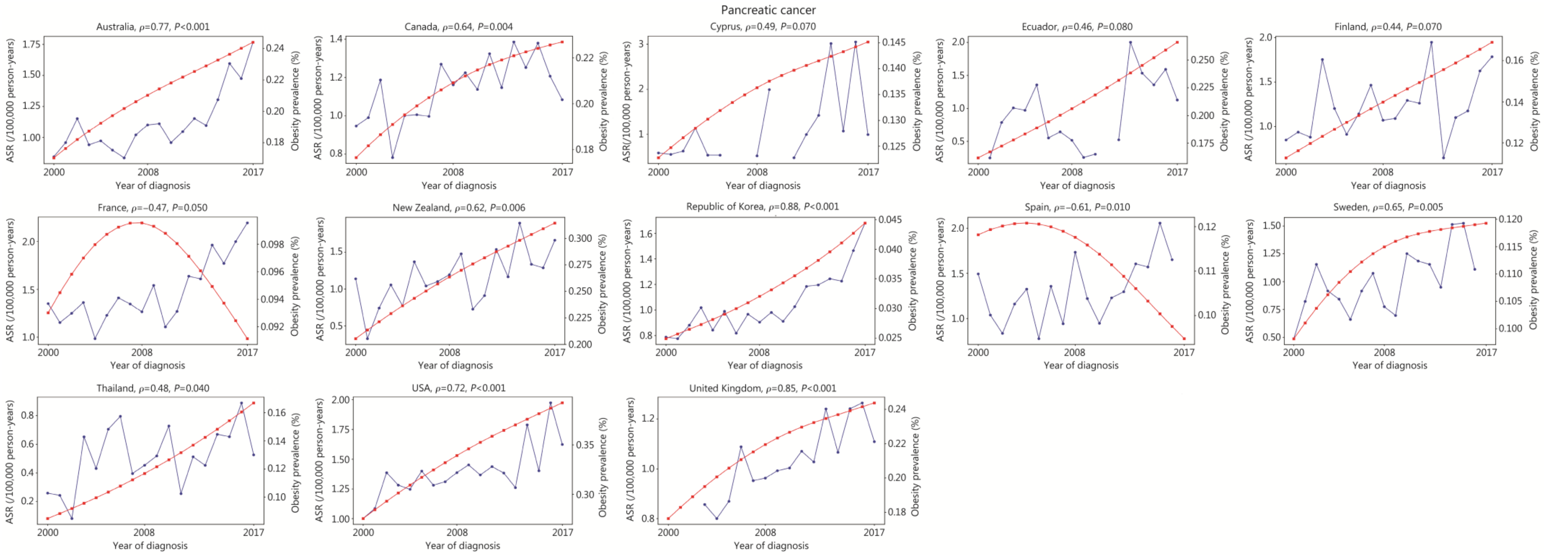

Fig. S6 Correlation between obesity prevalence and the incidence of early-onset pancreatic cancer among females. Blue lines show early-onset cancer incidence, and red lines show obesity prevalence among younger populations aged 20 – 49 years from 2000 to 2017. ASR age-standardized rate

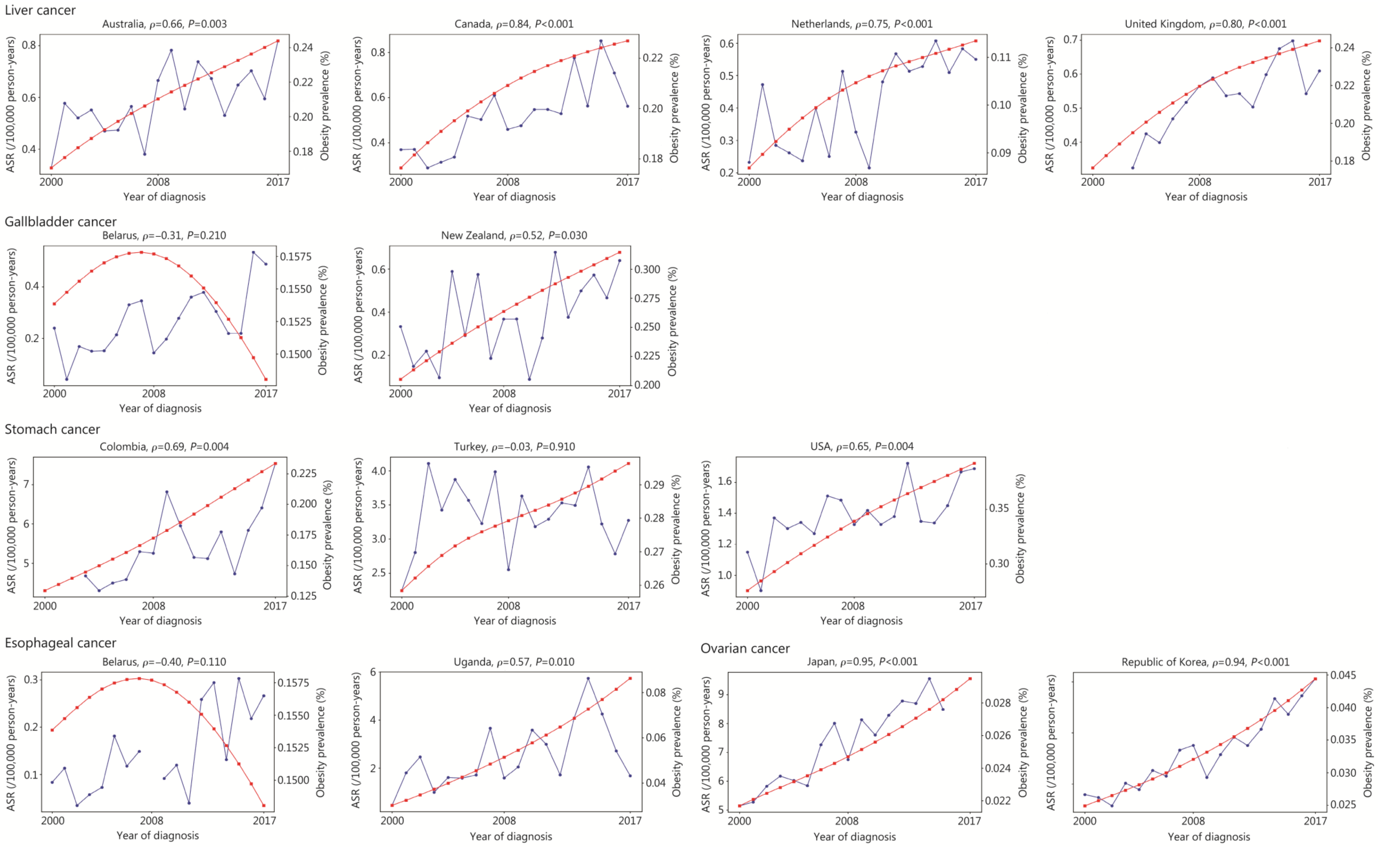

Fig. S7 Correlation between obesity prevalence and the incidence of early-onset liver, gallbladder, stomach, esophagus, and ovarian cancer among females. Blue lines show early-onset cancer incidence, and red lines show obesity prevalence among younger populations aged 20 – 49 years from 2000 to 2017. ASR age-standardized rate

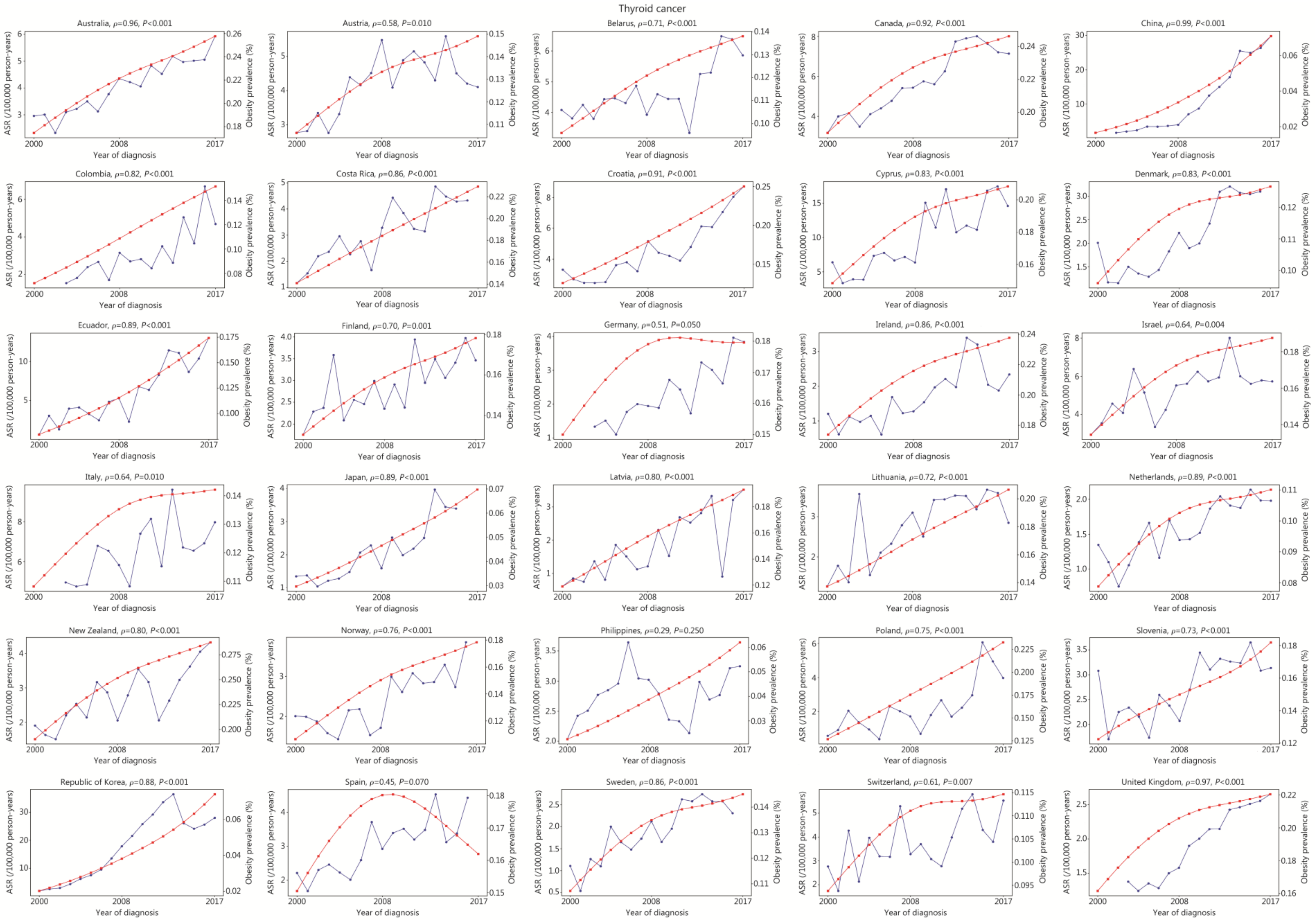

Fig. S8 Correlation between obesity prevalence and the incidence of early-onset thyroid cancer among males. Blue lines show early-onset cancer incidence, and red lines show obesity prevalence among younger populations aged 20 – 49 years from 2000 to 2017. ASR age-standardized rate

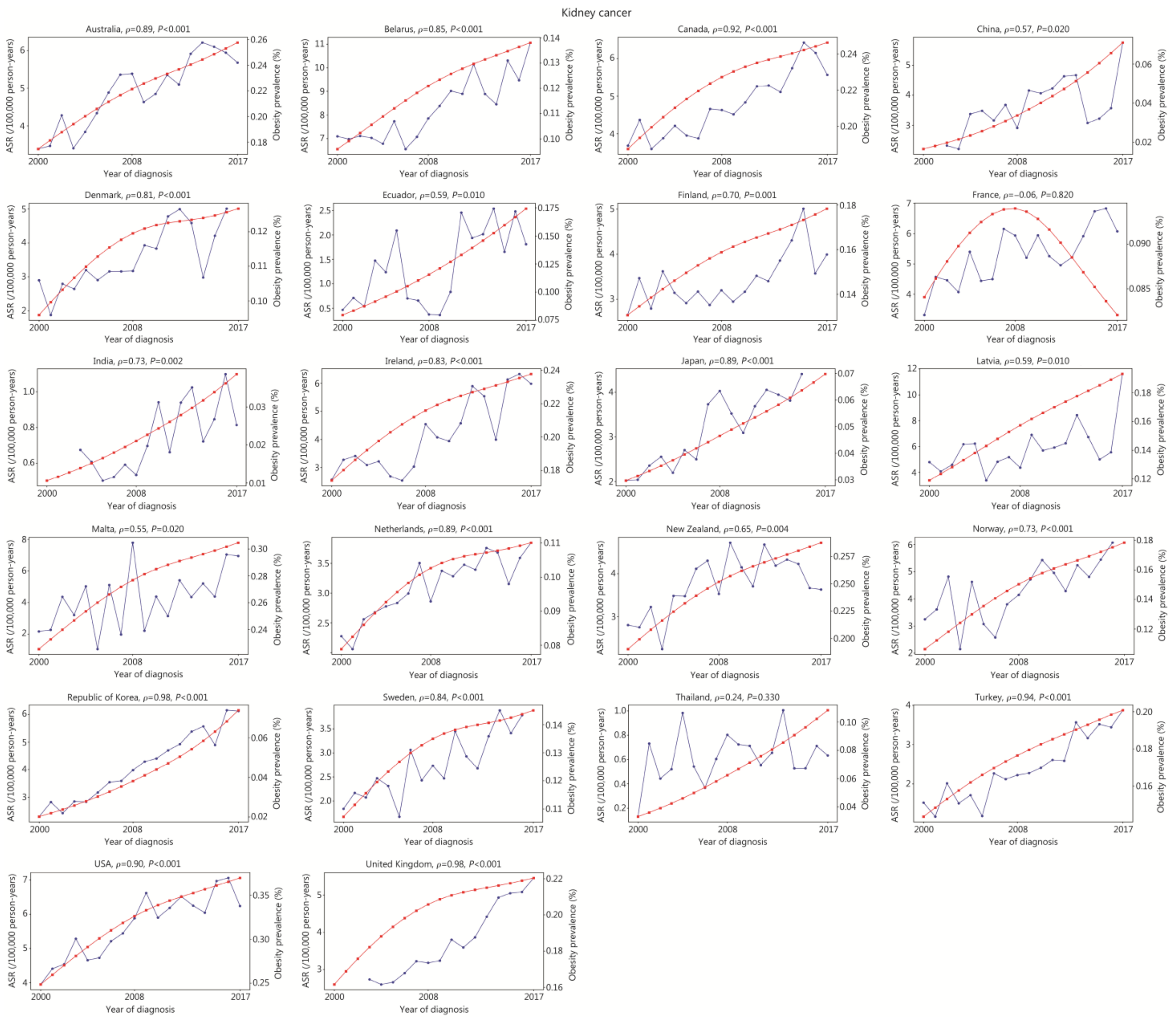

Fig. S9 Correlation between obesity prevalence and the incidence of early-onset kidney cancer among males. Blue lines show early-onset cancer incidence, and red lines show obesity prevalence among younger populations aged 20 – 49 years from 2000 to 2017. ASR age-standardized rate

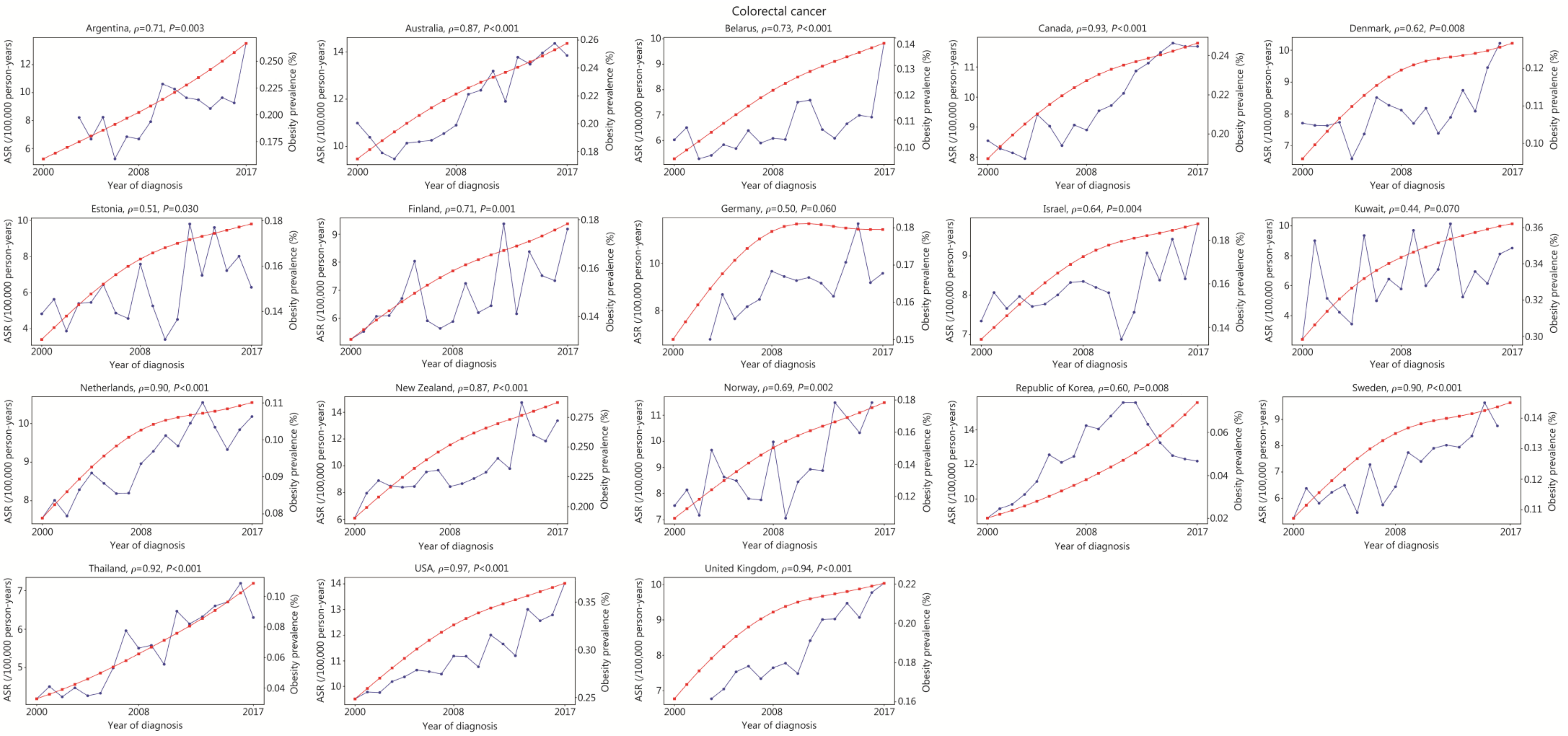

Fig. S10 Correlation between obesity prevalence and the incidence of early-onset colorectal cancer among males. Blue lines show early-onset cancer incidence, and red lines show obesity prevalence among younger populations aged 20 – 49 years from 2000 to 2017. ASR age-standardized rate

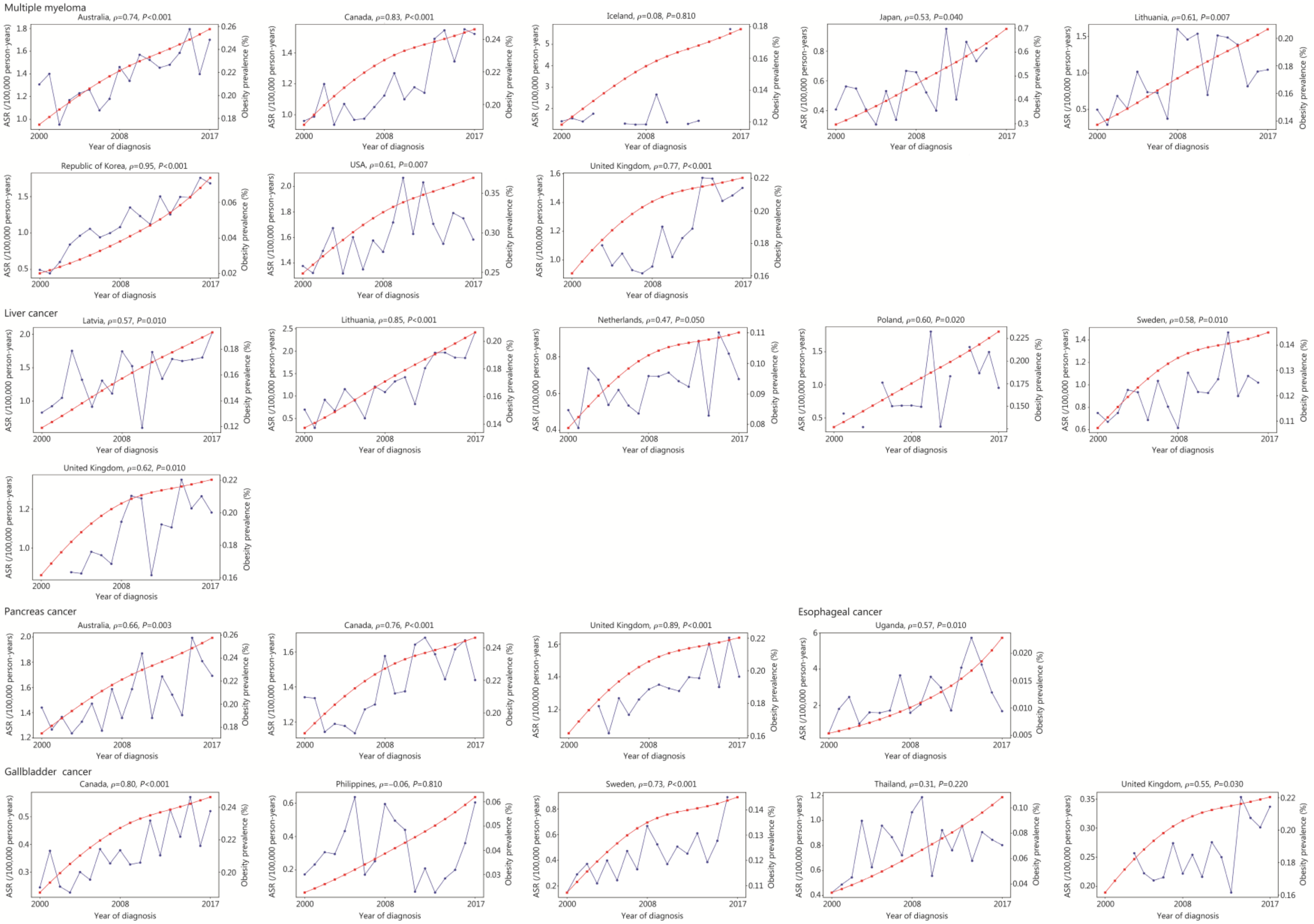

Fig. S11 Correlation between obesity prevalence and the incidence of early-onset multiple myeloma, liver cancer, pancreas cancer, esophagus cancer, and gall bladder cancer among males.

Blue lines show early-onset cancer incidence, and red lines show obesity prevalence among younger populations aged 20 – 49 years from 2000 to 2017. ASR age-standardized rate

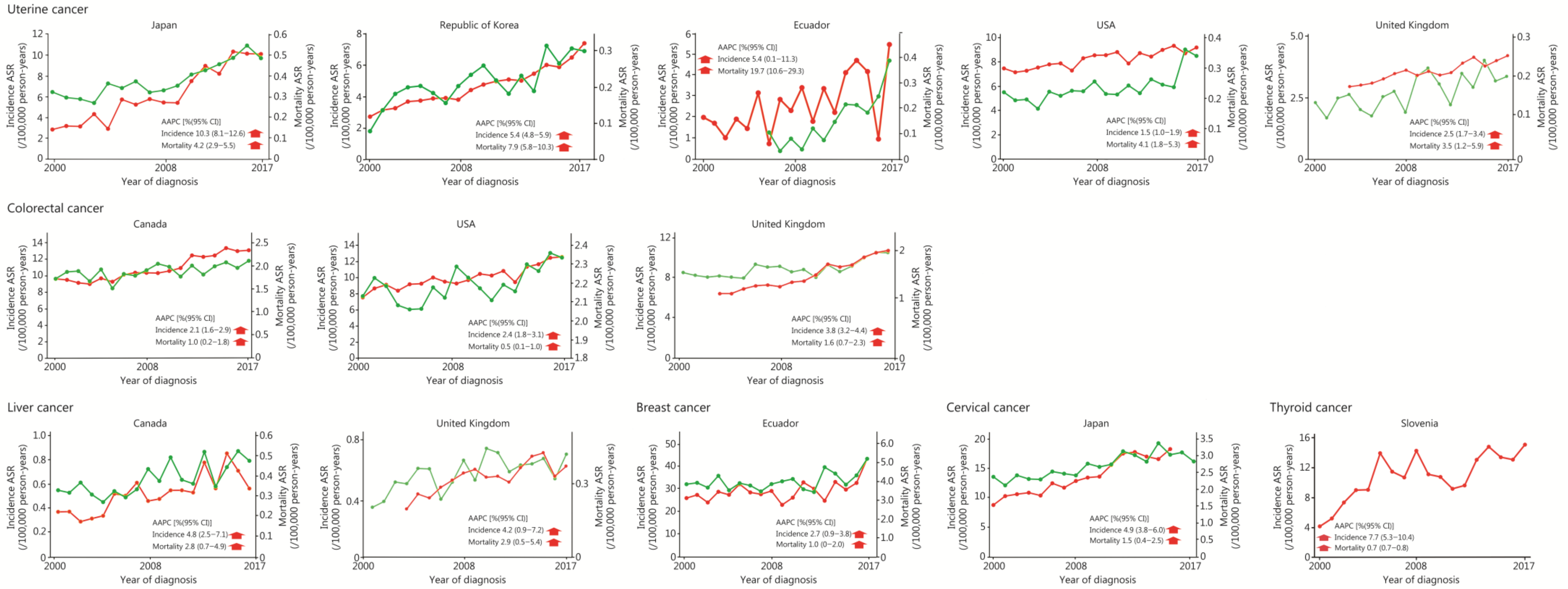

Fig. S12 Countries with increases in both incidence and mortality of early-onset cancers in females. Red lines show early-onset cancer incidence, and green lines show early-onset cancer mortality. Data on thyroid cancer in Sweden were not shown due to limited mortality data. AAPC average annual percentage change, ASR age-standardized rate

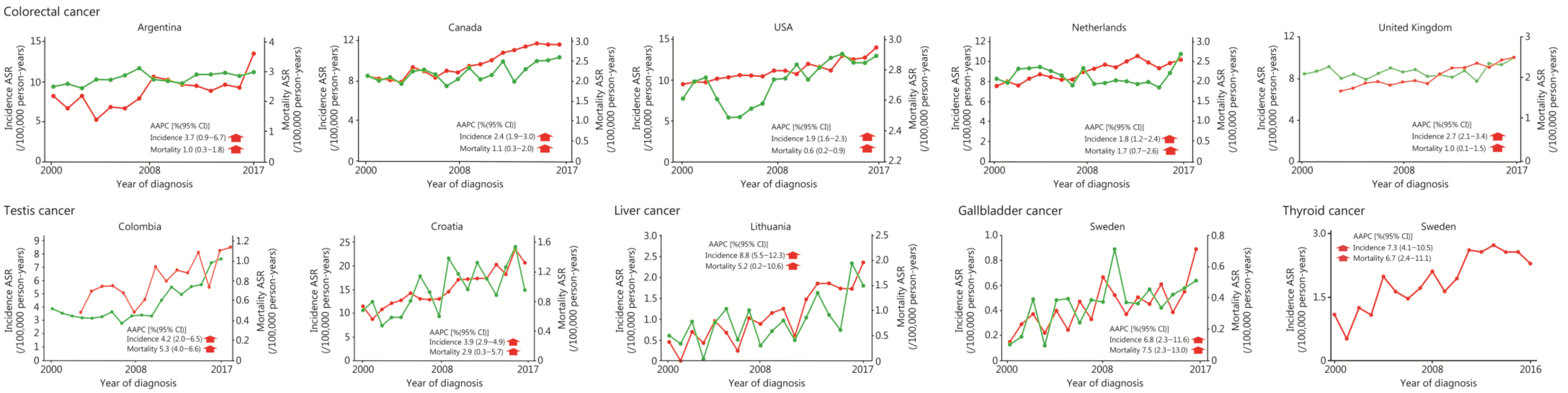

Fig. S13 Countries with increases in both incidence and mortality of early-onset cancers in males. Red lines show early-onset cancer incidence, and green lines show early-onset cancer mortality. Data on thyroid cancer in Sweden were not shown due to limited mortality data. AAPC average annual percentage change, ASR age-standardized rate

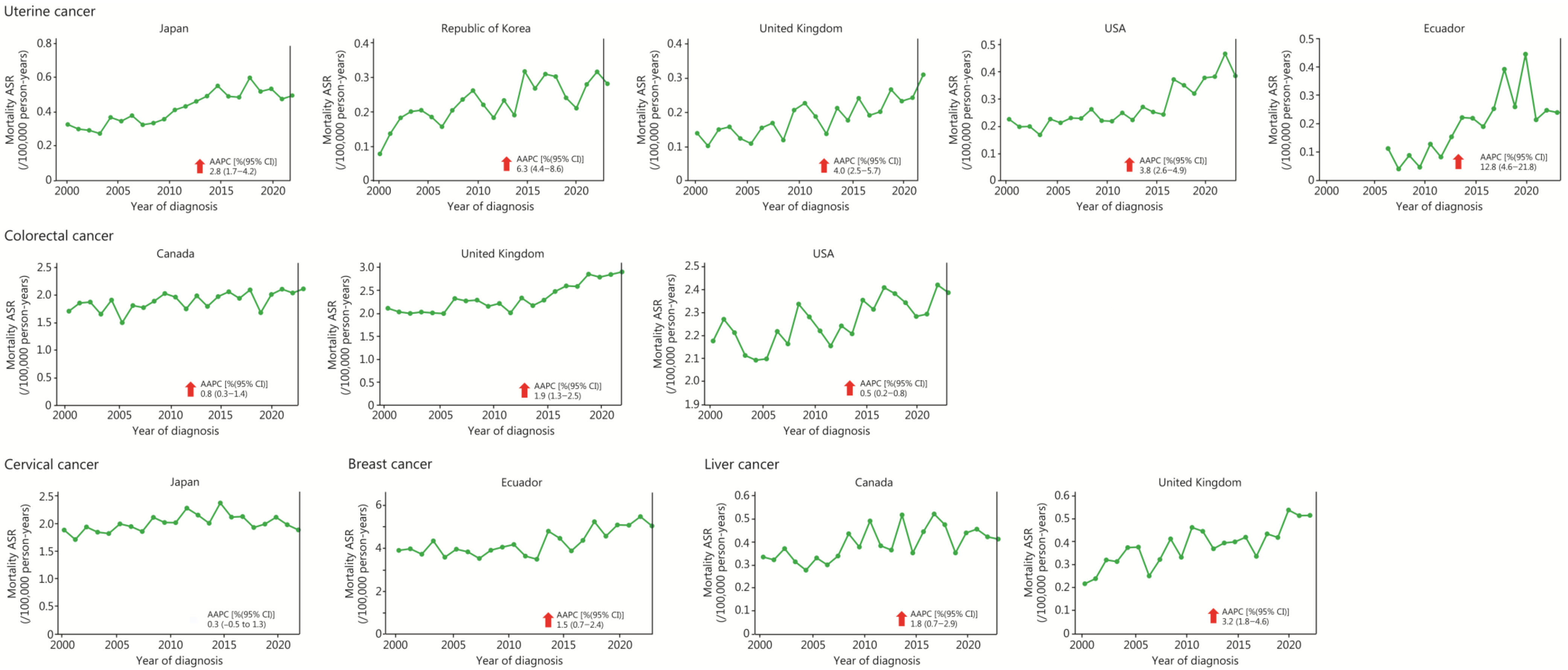

Fig. S14 Mortality trends of early-onset cancers from 2000 to 2023 (or 2021/2022 depending on data availability) in females. Data on thyroid cancer in Sweden were not shown due to limited mortality data. AAPC average annual percentage change, ASR age-standardized rate

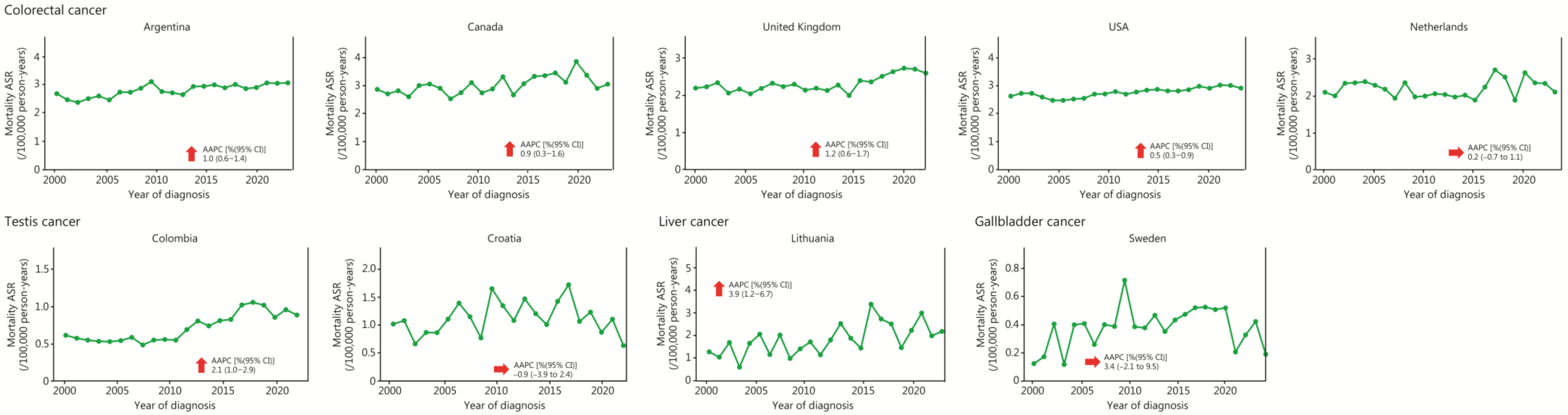

Fig. S15 Mortality trends of early-onset cancers from 2000 to 2023 (or 2021/2022 depending on data availability) in males. Data on thyroid cancer in Sweden were not shown due to limited mortality data. AAPC average annual percentage change, ASR age-standardized rate
